# Paravalvular mitral valve leakage presenting as congestive heart failure, missed by TTE but diagnosed by TEE: a case report

**DOI:** 10.1186/1757-1626-1-216

**Published:** 2008-10-06

**Authors:** Suriya Jayawardena, Danushan Sooriabalan, Olga Burzyantseva, Selvaratnam Sinnapunayagm

**Affiliations:** 1 Coney Island Hospital, 2601 Ocean Park Way, Brooklyn, NY 11235, USA

## Abstract

**Background:**

Diagnosis of prosthetic valve leakage by the transthoracic echocardiogram (TTE) technique is more difficult. These limitations are diminished with the use of transesophageal echocardiogram (TEE) techniques.

**Case report:**

A 71 year old Caucasian male presented with symptoms and signs of congestive heart failure. Past medical history included a bio-prosthetic mitral valve replacement for severe mitral regurgitation. TTE showed possible mitral regurgitation. As the TTE did not correlate with the finding of a high E-velocity, a TEE was performed, which showed a significant paravalvular leak of moderate severity around the bio-prosthetic mitral valve.

**Conclusion:**

There should be a high degree of suspicion to diagnose a paravalvular leak.

## Background

There is no perfect prosthetic heart valve. When selecting a valve for implantation, the advantages of each type must be weighed against its disadvantages. For example, mechanical valves have low rates of structural deterioration, but require the patient to be anticoagulated to avoid thromboembolic complications. Bio-prosthetic valves on the other hand have a short half life and are not preferred in younger patients [1]. Paravalvular regurgitation is an infrequent complication of valve replacement. It occasionally results from improper implantation of a valve, excessive calcification or friable and fragile tissues at the site of ring attachment due to infection [2].

With mild or moderate paravalvular leakage, patients may be asymptomatic and may have only a mild hemolytic anemia. They can be observed carefully with serial echocardiographic examinations [3]. Patients with severe paravalvular leakage usually have symptoms of heart failure or severe anemia and should be treated with surgical repair or replacement of the valve [4-6]. We present a patient who had mitral valve replacement due to sever mitral valve regurgitation presenting with heart failure three years later due to a paravalvular leak.

## Case report

A 71 year old male presented to the emergency department with shortness of breath and lower extremity swelling for one week. Physical examination revealed an afebrile, tachypneic man, the rest of his vital signs were within normal limits. The patient denied similar symptoms in the recent past after his valve replacement. On examination a left parasternal heave was felt, decreased breath sounds over both lung fields and an S1/S2 with a loud pulmonary component were auscultated. A holosystolic murmur graded 3/6 was heard over the mitral area.

**Figure 1:**
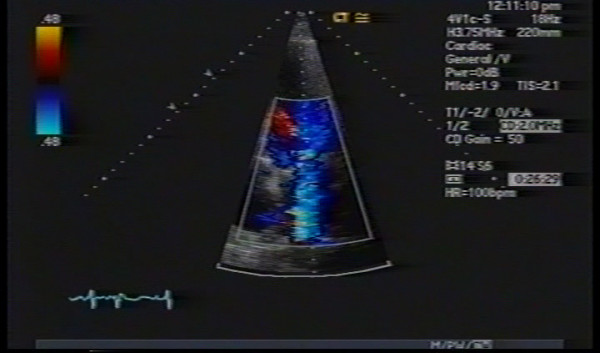
Transthoracic Echocardiogram showing (arrow) possible mitral valve regurgitation.

**Figure 2:**
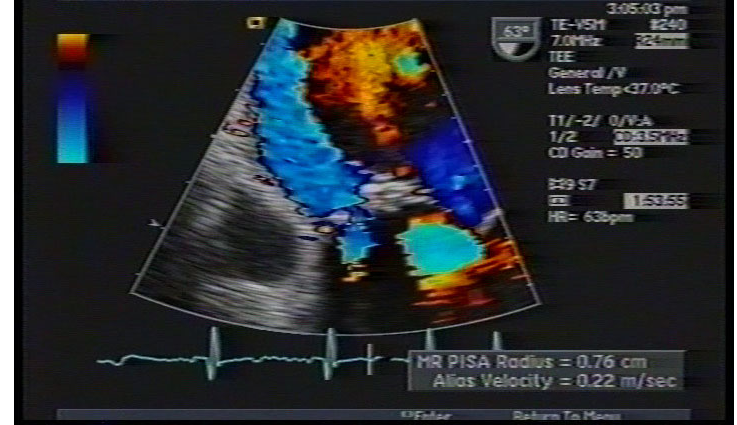
Transesophageal Echocardiogram showing (arrow) a paravalvular mitral valve leak missed by the transthoracic echocardiogram.

Past medical history included hypertension and atherosclerotic heart disease. He had a coronary artery bypass graft for triple vessel disease and a bio-prosthetic mitral valve replacement for severe mitral regurgitation. This prosthesis was subsequently replaced due to endocarditis three years ago. Preliminary labs did not show an increased white count or anemia, and the blood cultures were negative for growth.

(TTE) (Figure: 1) showed moderately decreased left ventricular systolic function with global hypokinesia and paradoxical septal motion consistent with open heart surgery, mild to moderate dilated left atrium, bio-prosthetic mitral valve with thickened leaflet, possible eccentric mitral regurgitation, pulmonary artery systolic pressure of 62 mm Hg, and a left ventricular ejection fraction of 30–35%. On Doppler interrogation E-velocity was high and color flow Doppler showed an eccentric mitral regurgitant jet. However as the assessment of the severity and the origin of the mitral regurgitation were not possible by TTE, a TEE (Figure: 2) was performed. The TEE showed a significant paravalvular leak of moderate severity around the bio-prosthetic mitral valve. Regurgitant orifice area of the paravalvular leak was 0.5 cm^2 ^by the PISA method, and regurgitant volume was 70 mL.

## Discussion

Cardiac auscultation remains the most widely used method of screening for heart disease. Heart murmurs are produced by turbulent blood flow and are often signs of stenotic or regurgitant valve disease that are acquired or congenital valve defects. When the characteristic findings of an individual murmur are considered together with other patient information and clinical data from the physical examination, the correct diagnosis can usually be established. In patients with ambiguous clinical findings, the echocardiogram may be the preferred test because it can provide a definitive diagnosis, rendering a chest radiograph and/or EKG unnecessary.

Delineation of normal prosthetic valve function is usually possible with TTE. Despite the prominent acoustic shadowing that accompanies prostheses, a Doppler signal that demonstrates normal transprosthetic flow velocity and flow duration is usually sufficient to exclude a stenotic valve, but exclusion of an incompetent valve is often more difficult, especially for prosthetic valves in the mitral position. Transthoracic Doppler echocardiography provides important hemodynamic information with regard to prosthetic valve pressure gradients and is extremely useful in long-term follow-up [3]. The "normal gradient" across a prosthetic valve depends upon the type, size, and position of the prosthesis as well as the cardiac output; guidelines are available for the acceptable range of Doppler gradients encountered in properly functioning valves [7,8]. See Table 1.

**Table 1:** Accepted mean pressure gradients across aortic and mitral prosthetic valves.

Valve type	Aortic		Mitral		
	V max (m/s)	Mean Gradient (MG) (mmHg)	V max (m/s)	Mean Gradient(MG) (mmHg)	Causes of increase MG
Mechanical					
Bileaflet (St. Jude)	2.5	12	1.6 +/- 0.3	4 +/- 1	Hyperdynamic Ventricle, Paravalvular leak, Suture déhiscence/abscess
Tilting disk	2.6 +/- 0.4	14 +/- 5	1.6 +/- 0.3	3 +/- 2	
Ball cage	3.1 +/- 0.5	24 +/- 4	1.9 +/- 0.5	5 +/- 2	
Tissue valves					
Stented	2.6 +/- 0.4	13 +/- 6	1.8 +/- 0.2	6 +/- 2	Valve regurgitation, endocarditis, thrombosis
Non stented	2.2 +/- 0.4	3 (2–20)	1.5 +/- 0.2	4 +/- 2	
AO homograft	1.8 +/- 0.4	7 +/- 3			

The V_max _and mean gradients across the aortic and mitral valves mentioned in the table are the normal pressures gradients expected in a size 23 mm prosthetic valve.[14]

Acoustic shadowing caused by the prosthetic material may limit transthoracic visualization of prosthetic leaflets. Prosthetic aortic valve regurgitation is usually well visualized on surface color Doppler imaging but prosthetic mitral regurgitation is frequently undetectable[9]. As in our patient, TEE is the imaging modality of choice when the TTE is suboptimal or when there are borderline findings on the TTE in a patient whom there is a strong clinical suspicion of malfunction [8]. The overall sensitivity and specificity for TTE is 57% and 63% respectively and for TEE it was 86% and 88% for the identification of morphological abnormalities of prosthetic valves [10]. In another study it was reported that the sensitivity for the detection of paravalvular leaks for mitral and aortic valves by TTE was 25% and 45% respectively where as by TEE it was 100% [11].

To recognize a paravalvular leak, TEE must be performed with a high color frame rate and middle range Nyquist limits (35 to 50 cm/sec) in several views from several angles outside the sewing ring [12]. The echocardiographer should do a careful search for periprosthetic leaks around as much of the valve circumference as possible and attempt to define the extent of the regurgitation once it is identified. The origin of a periprosthetic leak may appear deceivingly narrow when it is related to disruption of a limited number of sutures. In some cases, these jets are seen inadvertently when one examines other structures, such as the interatrial septum. The most severe form of paravalvular regurgitation is seen when there is dehiscence of a substantial portion of the sewing ring [13]. In this setting, there is severe regurgitation, such that the regurgitant flow is almost laminar. As in angiography, the sewing ring on echocardiography is seen to rock with each cardiac cycle; mobile echo densities representing the liberated suture material often can be visualized. Both the laminar flow and the rocking motion of the ring may elude the inexperienced echocardiographer, thus delaying recognition of this life threatening condition [13]. The physician evaluating a prosthetic device with TEE should be aware of the range of abnormalities that are possible in these devices and should match those possibilities to the patient's presentation.

## Conclusion

Paravalvular leak can occur as in our patient without endocarditis few years after the valve replacement. As in our patients who presented with a life threatening heart failure, the physician and the echocardiographer should have a high degree of suspicion in diagnosing and treating paravalvular leaks.

## Abbreviations

TTE: Transthoracic echocardiogram; TEE: Transesophageal echocardiogram; PISA: Proximal isovelocity surface area; EKG: Electrocardiogram.

## Consent

A written informed consent was obtained from the patient for publication of this case report and accompanying images. A copy of the written consent will be made available on request

## Competing interests

The above case report was written at Coney Island Hospital. The above mentioned authors have no affiliation to any other institute other than Coney Island Hospital.

## Authors' contributions

SJ, DS and OB treated the patent and were responsible for writing the paper and looking up the back ground references. SS was responsible for over all coordination and final proof reading. All the above mentioned authors read and approved the final manuscript.
